# ^90^Y-NM600 targeted radionuclide therapy induces immunologic memory in syngeneic models of T-cell Non-Hodgkin’s Lymphoma

**DOI:** 10.1038/s42003-019-0327-4

**Published:** 2019-02-26

**Authors:** Reinier Hernandez, Kirsti L. Walker, Joseph J. Grudzinski, Eduardo Aluicio-Sarduy, Ravi Patel, Christopher D. Zahm, Anatoly N. Pinchuk, Christopher F. Massey, Ariana N. Bitton, Ryan J. Brown, Paul M. Sondel, Zachary S. Morris, Jonathan W. Engle, Christian M. Capitini, Jamey P. Weichert

**Affiliations:** 1 Department of Radiology, University of Wisconsin School of Medicine and Public Health, Madison, WI USA; 2 Department of Pediatrics, University of Wisconsin School of Medicine and Public Health, Madison, WI USA; 3 Department of Medical Physics, University of Wisconsin School of Medicine and Public Health, Madison, WI USA; 4 Department of Human Oncology, University of Wisconsin School of Medicine and Public Health, Madison, WI USA; 5 University of Wisconsin Carbone Cancer Center, University of Wisconsin School of Medicine and Public Health, Madison, WI USA

## Abstract

Finding improved therapeutic strategies against T-cell Non-Hodgkin’s Lymphoma (NHL) remains an unmet clinical need. We implemented a theranostic approach employing a tumor-targeting alkylphosphocholine (NM600) radiolabeled with ^86^Y for positron emission tomography (PET) imaging and ^90^Y for targeted radionuclide therapy (TRT) of T-cell NHL. PET imaging and biodistribution performed in mouse models of T-cell NHL showed in vivo selective tumor uptake and retention of ^86^Y-NM600. An initial toxicity assessment examining complete blood counts, blood chemistry, and histopathology of major organs established ^90^Y-NM600 safety. Mice bearing T-cell NHL tumors treated with ^90^Y-NM600 experienced tumor growth inhibition, extended survival, and a high degree of cure with immune memory toward tumor reestablishment. ^90^Y-NM600 treatment was also effective against disseminated tumors, improving survival and cure rates. Finally, we observed a key role for the adaptive immune system in potentiating a durable anti-tumor response to TRT, especially in the presence of microscopic disease.

## Abstract (web summary)

Hernandez et al. show the effectiveness of ^90^Y for targeted radionucleotide therapy of T-cell Non-Hodgkin’s Lymphoma (NHL). This study suggests that delivering radiation to all NHL disease sites elicits minimal toxicity and induces a memory T-cell response, inviting combination therapies with immune activating agents.

## Introduction

T-cell non-Hodgkin lymphomas (NHL) are a heterogeneous group of diseases that account for 10 to 15% of all lymphomas in the US. High-risk T-cell NHL is associated with poor prognosis. Reported 5-year survival rates after standard of care treatment are highly dependent upon disease pathology and staging but can be as low as 11% in some adult T-cell leukemias/lymphomas (ATLL)^1,2^. Therefore, novel therapeutic strategies/combinations are urgently needed for patients with T-cell NHLs. Immunoconjugates including monoclonal antibodies (mAb) and mAb-drug conjugates targeting CD30, CD52, CD4, and chemokine receptor 4 (CCR4); histone deacetylase (HDAC) inhibitors; antifolates; fusion proteins; nucleoside analogs; and several other combination regimens have been evaluated for the treatment of T-cell NHL^2^. Despite these efforts, the improvements on T-cell NHL mortality have been modest, and further studies are needed^3,4^. Localized external beam radiation therapy (EBRT) achieves elevated response rates and can be curative for early stage T-cell NHL^5,6^, but for stage III/IV disseminated disease, response rates with chemotherapy and radiation are still poor^7^.

A systemic approach that delivers radiation doses to disseminated disease may have a tremendous impact on the treatment of advanced stage T-cell NHL. In that regard, targeted radionuclide therapy (TRT) can selectively deliver lethal radiation doses to tumor cells while sparing normal tissues. Such an approach has been proven effective in treating relapsed/refractory B-cell lymphomas in which a single dose administration of ^131^I-tositumomab (Bexxar) or ^90^Y-ibritumomab (Zevalin), two radiolabeled anti-CD20 chimeric mAbs, afforded high response (47-68%) rates within heavily pretreated populations^8–12^. Due to the expression of CD20 on both malignant and normal B-cells, clinicians first administer cold mAb to saturate and protect normal B-cells, followed by radiolabeled mAb to target tumor cells. Because the secondary hypogammaglobulinemia from B-cell aplasia can be treated with intravenous immunoglobulin, this approach is feasible for B-cell NHL. However, in T-cell NHL, the secondary T-cell aplasia cannot be treated leading to profound risk for infection and tumor relapse. Thus, due to the relative scarcity of targets that are specific to T-cell NHL and not normal T cells, TRT paradigms are yet to be implemented for the treatment of T-cell NHL.

Here we exploit the ability of cancer cells to selectively sequester and retain alkylphospholipids compare to normal cells to develop a radiolabeled alkylphosphocholine (APC) analog as a TRT agent for the treatment of murine models of T-cell NHL^13–15^. Using a theranostic approach, NM600, an APC analog featuring a DOTA chelator is labeled with the positron emitter ^86^Y, for noninvasive PET/CT imaging to assess the tumor targeting characteristics and predict the efficacy of ^90^Y-NM600. The differential uptake of ^90^Y-NM600 in malignant T-cells provides an excellent therapeutic window that allows high curative rates in both localized and disseminated models of this disease, with no relevant toxicities. Additionally, we demonstrate the interplay of TRT with the innate immune system in obtaining an elevated degree of complete response. To our knowledge, this work represents the first successful implementation of a TRT approach for the treatment of T-cell NHL.

## Results

### Radiochemistry and in vitro uptake

**Fig. 1:**
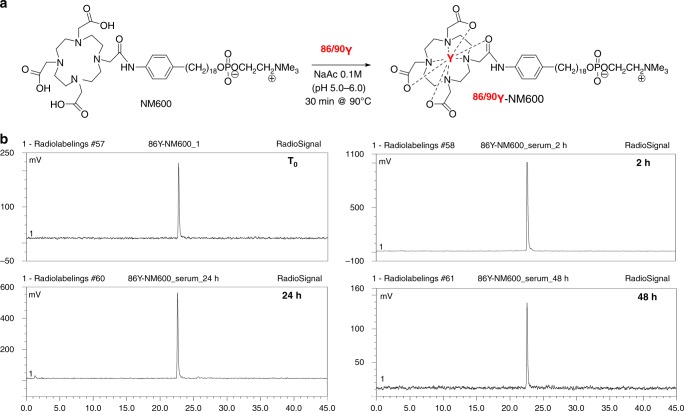
Radiochemical synthesis and stability of ^86^Y/^90^Y-NM600. **a** Schematic representation of the radiochemical synthesis of ^86^Y/^90^Y-NM600. **b** Stability of ^86^Y-NM600 in mouse serum for up to 48 h was determined by radio-HPLC. Radiochromatograms at each timepoint shows no indications of NM600 degradation or ^86^Y transchelation

Radiolabeling of NM600 (Fig. 1a) with ^86^YCl_3_ or ^90^YCl_3_ was achieved quantitatively with yields and radiochemical purity consistently surpassing 95%, as determined by iTLC and radio-HPLC. A similar average molar specific activity of 18 GBq/µmol was obtained for both ^86^Y-NM600 and ^90^Y-NM600 (*n* > 5). Radiotracer stability in mouse serum up to 48 h was analyzed by radio-HPLC. No significant radiopeaks corresponding to metabolites were observed in any of the longitudinal chromatograms indicating the excellent serum stability of the compound (Fig. 1b). The selectivity of ^86^Y-NM600 for cancerous EL4 cells compared to normal T-cells was determined in vitro. Incubation of ^86^Y-NM600 with EL4 cells and T-cells isolated from the spleen of naïve wild-type mice showed a 4-fold preferential accumulation of the compound in malignant T-cells (Supplementary Figure 1).

### Longitudinal in vivo PET/CT imaging and biodistribution

**Fig. 2:**
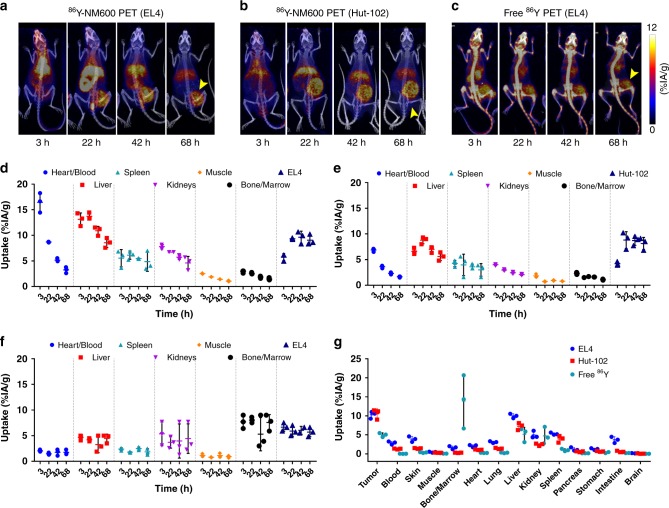
PET/CT imaging and biodistribution of ^86^Y-NM600 in mice bearing T-cell NHLs. Representative maximum intensity projections (MIP) of PET/CT images showing prominent and persistent tumor uptake in mice bearing EL4 (**a**) or Hut-102 (**b**) injected 9.25 MBq ^86^Y-NM600. Free ^86^Y (**c**) injection in EL4 bearing mice showed marked uptake in bone but lower accumulation in EL4 tumors. Yellow arrowheads point to the tumor. ROI quantitative analysis of ^86^Y-NM600 (**d**, **e**) and free ^86^Y (**f**) biodistribution in EL4 and Hut-102 tumor-bearing mice. **g** Ex vivo biodistribution was performed after the final imaging time point 68 h p.i. Numerical data are presented as %IA/g (mean ± SD)

Longitudinal PET/CT imaging was performed to elucidate the in vivo tumor targeting and biodistribution of ^86^Y-NM600 and to identify potential risk organs for radiotoxicity. Figure 2 shows representative maximum intensity projection (MIP) PET/CT images acquired at 3, 24, 48, and 72 h post-injection of ^86^Y-NM600 in mice bearing subcutaneous (s.c.) syngeneic murine EL4 (Fig. 2a) or xenogeneic human Hut-102 (Fig. 2b) T-cell NHL xenografts. Additionally, another cohort of EL4 bearing mice was administered free ^86^Y (Fig. 2c) to demonstrate the tumor specificity of ^86^Y-NM600. It is evident from the PET/CT images that there is an elevated and persistent accumulation of ^86^Y-NM600 in both the murine and human NHLs affording clear tumor conspicuity as early as 24 h post-injection. On the other hand, a notably different in vivo distribution profile was observed for unchelated free ^86^Y, which exhibited lower tumor uptake and marked deposition in bone. This distribution of the free radiometal is in accordance with previous reports showing accumulation of Yttrium isotopes in the liver, spleen, and the bones of normal animals, across several species^16,17^.

^86^Y-NM600 uptake in the tumor and normal tissues (%IA/g) was quantified via a region-of-interest (ROI) analysis of the PET/CT images at each time point which generated time-activity curves (TAC) which can be used for pharmacokinetic analysis and dosimetric calculations. A high accumulation in the blood pool (“heart/blood”) of 16.45 ± 1.91 %IA/g and 6.87 ± 0.32 %IA/g was observed initially in mice bearing either EL4 or Hut-102 tumors, respectively (*n* = 3) (Fig. 2d, e). Monoexponential fitting of the heart TAC indicated similar ^86^Y-NM600 blood kinetics in both groups, with relatively long circulation half-lives of 17.26 ± 4.57 h and 15.33 ± 2.13 h, in EL4 and Hut-102 bearing mice, respectively **(**Supplementary Figure 2**)**. ^86^Y-NM600 accumulation in the liver (Fig. 2, Supplementary Table 1), which reached peak uptake values of 13.73 ± 0.64 %IA/g and 8.77 ± 0.68 %IA/g, in EL4 and Hut-102 groups at 24 h p.i., gradually declined, suggesting hepatobiliary excretion of the tracer. In normal tissues including the spleen, kidneys, bone/marrow, and muscle, ^86^Y-NM600 accretion displayed lower initial values that gradually decreased to background levels at later timepoints. In contrast, injection of free ^86^Y resulted in a rapid distribution of the activity to kidneys, liver, and more prominently, bone, with values that remained persistently high for up to 72 h p.i. (Fig. 2, Supplementary Table 1) and demonstrated limited excretion of free ^86^Y.

EL4 and Hut-102 tumors both exhibited increasing ^86^Y-NM600 accumulation (5.74 ± 0.68 %IA/g vs. 4.20 ± 0.36 %IA/g at 3 h p.i.; *n* = 3) reaching plateau values of 9.35 ± 0.15 %IA/g and 8.80 ± 1.64 %IA/g at 24 h p.i., respectively. Owning to prolonged radioactivity retention, the tumor presented the highest %IA/g values of all tissues at 72 h post ^86^Y-NM600 administration. Interestingly, unchelated ^86^Y also accumulated within the tumor, but to a significantly lower extent (*P* < 0.001) and with markedly different kinetics (Fig. 2f).

Ex vivo biodistribution was performed after the terminal PET/CT scan, 72 h p.i., to corroborate the imaged-based quantitative data and to determine more comprehensive tissue distribution of ^86^Y-NM600 in normal tissue **(**Fig. 2g and Supplementary Table 2**)**. Broad agreement was observed between quantitative PET/CT data and ex vivo biodistribution in both magnitude and trends. EL4 and Hut-102 tumors displayed the highest ^86^Y-NM600 uptake of all tissues (10.28 ± 0.74 %IA/g vs. 10.54 ± 1.08 %IA/g) followed by the liver (9.98 ± 0.47 %IA/g vs. 7.26 ± 0.76 %IA/g); uptake in the remaining tissues was less pronounced ( < 5 %IA/g). Consistent with the imaging data, biodistribution of non-chelated ^86^Y was distinct from that of ^86^Y-NM600, with bone, liver, and kidneys showing the high radioactivity accumulations of 13.90 ± 5.72 %IA/g, 5.14 ± 1.48 %IA/g, 4.72 ± 1.80 %IA/g, respectively. Uptake of the free metal in EL4 tumors was approximately 50% lower compared to ^86^Y-NM600 (4.98 ± 0.42 %IA/g vs. 10.28 ± 0.74 %IA/g).

### ^90^Y-NM600 dosimetry estimation

^90^Y-NM600 tumor and normal organ dosimetry was estimated using PET/CT-derived time-activity curves and applying The Standard Mouse Model. Supplementary Table 3 summarizes the results of the dosimetry calculation showing an integral absorbed dose per activity of 2.69 Gy/MBq to EL4 tumors. Of the normal tissues, only the liver and the lungs showed similarly high absorbed doses of 3.04 and 3.34 Gy/MBq, respectively. The artificially elevated lung doses are the result of two phenomena^18,19^. First, the relatively long positron range of ^86^Y cause signal spillover into the lungs from the heart and liver which affect the quantification of PET images, resulting in the overestimation of the activity present in this organ. Secondly, due to the proximity of the liver and heart to the lungs, disintegrations that originate in the liver and heart often extend into the lungs due to the long range of the β- emissions of ^90^Y. The remaining normal tissues exhibited absorbed dose values well under 2.0 Gy/MBq.

### Normal tissue toxicity assessment

**Fig. 3:**
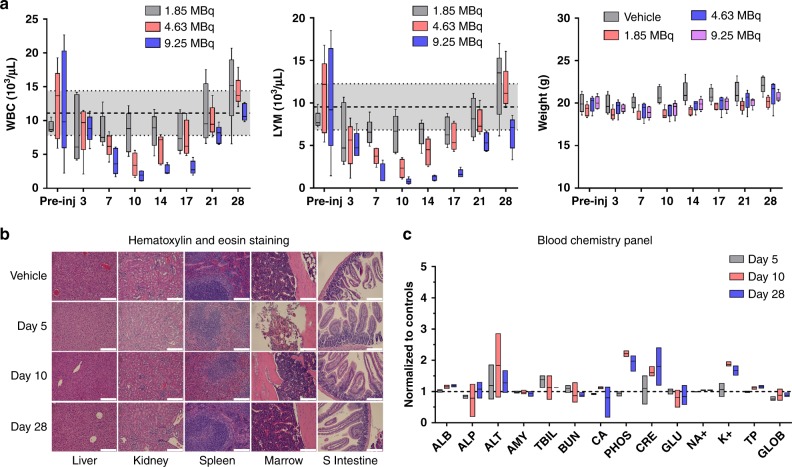
Tocixity assesmment in naïve B6 mice administered ^90^Y-NM600. **a** Longitudinal CBC panel and weight monitoring in mice injected 1.85, 4.63, or 9.25 MBq ^90^Y-NM600 IV. Mild, dose-dependent cytopenias were observed with a NADIR at day 10 p.i. and a three-week recovery period. Shaded areas represent the 95% CI of the normal values. No significant variation in weight was recorded in any of the different dose cohorts. **b** H&E staining of potential organs at risk was carried out in mice given the highest ^90^Y-NM600 IA (9.25 MBq) at days 5, 10, and 28 p.i. (*n* = 3). Day 5 shows a reduction in cellularity in the spleen white pulp and the bone marrow, which where largely recovered at day 10. Scale bar represents 100 μm. **c** Longitudinal panel of serum biomarkers indicative of systemic toxicity in mice injected 9.25 Mbq ^90^Y-NM600 (*n* = 3)

A comprehensive set of studies were carried out to interrogate potential acute toxicities associated with the administration of ^90^Y-NM600. Figure 3a (Supplementary Table 4-10**)** shows an activity escalation study in which naïve B6 mice were administered 1.85, 4.63, or 9.25 MBq of ^90^Y-NM600 (*n* = 5) and CBC and animal weight regularly monitored over a period of 28 days. No significant variation in mouse weight was observed at any activity level during the observation period. Mice given 4.63 or 9.25 MBq ^90^Y-NM600 injections experienced activity-dependent signs of bone marrow toxicity evidenced by significant (*P* *<* 0.0001) reductions of hematological parameters including white blood cells, lymphocytes, red blood cells, and hemoglobin with a NADIR at day 10 post-injection. Even at the highest activity level, the effects were mild and did not result in animal lethality, resolving within three weeks of injection. Another cohort of mice was administered 9.25 MBq ^90^Y-NM600, sacrificed at days 5, 10, and 28 (*n* = 3) after injection, and blood, liver, kidneys, spleen, bone marrow, and small intestine tissues collected for histopathology and blood chemistry analysis (Fig. 3b, c, Supplementary Table 11). Concurrent with the transient cytopenia revealed by CBC, H&E staining of bone marrow and spleen tissue unveiled notably reduced cellularity at day 5 p.i. that preceded the systemic reduction in the corresponding hematologic values. Normal bone marrow morphology recovered by day 10, whereas spleens were normal at day 28. No evidence of morphological changes were observed in liver, kidneys, and intestine after treatment. Finally, a blood chemistry panel including 14 parameters indicative of kidney and liver toxicity, revealed a slight increase in blood levels of creatinine, phosphate, and potassium that were not significant (*n* = 3; *P* > 0.05) compared to untreated controls (Fig. 3c). Overall, these results demonstrated that ^90^Y-NM600 at activities up to 9.25 MBq were below a maximum tolerable activity (MTA) level, owing to the minor toxicities associated with the administration of the TRT agent.

### ^90^Y-NM600 TRT in localized disease

**Fig. 4:**
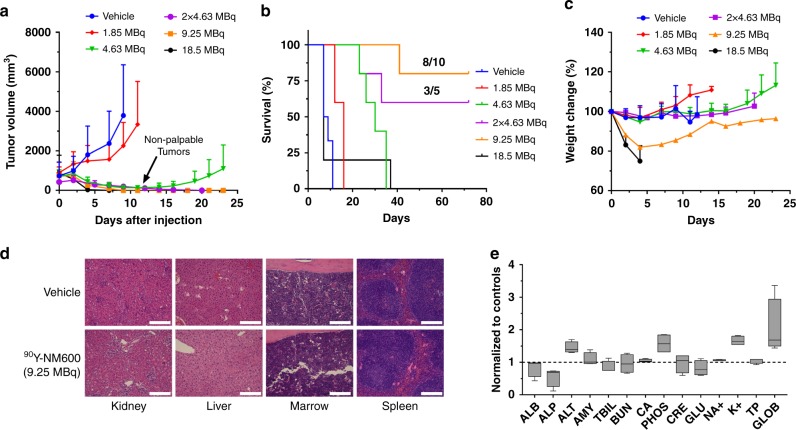
^90^Y-NM600 TRT achieves complete tumor responses with a favorable safety profile. **a** Growth of EL4 tumor after treatment with increasing IA of ^90^Y-NM600. An IA-dependent tumor growth inhibition was noted with complete tumor regression in the groups administered 2 × 4.63 MBq and 9.25 MBq of activity. **b** Overall survival. A significant improvement in survival was achieved in all treatment arms except the 18.5 MBq cohort; however, mice administered ^90^Y-NM600 2 × 4.63 MBq or 9.25 MBq exhibited complete responses (CR) in 60% (3/5) and 80% (8/10) of the subjects, respectively. **c** Body weight of treated mice. A noticeable decrease in body weight was observed in the 9.25 MBq and 18.5 MBq IA groups. **d** Normal tissue histology at day 150 after treatment. H&E staining of tissues slides of the kidneys, liver, bone marrow, and spleen shows normal morphology in CR mice compared to vehicle-treated controls. Scale bar represents 100 μm. **e** Comprehensive blood chemistry panel. No statistical difference was found in any of the blood markers compared to vehicle-treated controls. All numerical data are presented as mean ± SD

An injected activity-dependent tumor response was observed in all cohorts of mice bearing s.c. EL4 tumors injected intravenously with ^90^Y-NM600 (Fig. 4a and Supplementary Figure 3). Mice given 1.85 MBq (50 µCi; *n* = 5) ^90^Y-NM600 experienced a slight decrease in tumor growth rate but failed to achieve significance at any given time point (*P* > 0.07) compared to the vehicle group (*n* = 8). On the other hand, single 4.63 MBq (125 µCi; *n* = 5), 9.25 MBq (250 µCi; *n* = 2 × 5), or 18.5 MBq (500 µCi; *n* = 5) IV injections of ^90^Y-NM600 resulted in complete tumor regression (*P* < 0.001), 10 days after administration. Tumor regrowth occurred at the primary site in the 4.63 MBq group suggesting an incomplete eradication of the EL4 tumors cells. However, when mice received two fractionated injections of 4.63 MBq, 10 days apart, local control of the primary tumor was achieved. Despite achieving complete tumor eradication, 80% of the animals in the 18.5 MBq cohort were removed from the study on day 7 due to significant weight loss or cachexia (Fig. 4c).

Except in mice treated with 18.5 MBq ^90^Y-NM600, which succumbed to radiation toxicity, prolonged survival was achieved in all treatment arms compared to controls (Fig. 4b; *P* < 0.0001). Median survival of 8, 16, 30, and 7 days was recorded for the vehicle, 1.85 MBq, 4.63 MBq, and 18.5 MBq groups, respectively. A large fraction of animals in the 9.25 or 2 × 4.63 MBq groups exhibited complete response (CR) – 8 of 10, and 3 of 5 animals were tumor free at day 70 post-treatment, respectively. Of note, lethality in these cohorts was due to tumor recurrence and/or progression in either the primary or a distant site, commonly the axillary lymph nodes.

### ^90^Y-NM600 treatment toxicity evaluation

Single or fractionated injections up to 4.63 MBq had minimal effects on mouse weight (Fig. 4c Supplementary Figure 4). However, animals in the 9.25 and 18.5 MBq treatment groups showed a notable decline in body weight that resulted in euthanasia of the 18.5 MBq cohort ( > 20% weight loss) at day 5 after injection. Mice administered 9.25 MBq ^90^Y-NM600 on the other hand, experienced a steady recovery over a 3-week period.

Having already established the short-term toxicity profile of ^90^Y-NM600 and determining that it is tolerable up to 9.25 MBq with respect to acute effects, we wanted to investigate the long-term toxicity of ^90^Y-NM600. To that end, we sacrificed half of the complete responders in the 9.25 MBq group (*n* = 4) at day 150 after initial treatment and performed histopathological analysis of the kidneys, liver, spleen, and bone marrow, as well as a CBC and chemistry analysis of the blood (Supplementary Table 4-11). H&E staining showed no evidence of organ damage as tissue morphology was similar between the treated and control subjects in all cases (Fig. 4d). Moreover, the results of the CBC and blood chemistry analysis were within normal values compared to age-matched controls. These results suggested a favorable toxicity profile, both short-term and long-term, at or below this injected activity.

### ^90^Y-NM600 TRT in systemic disease

**Fig. 5:**
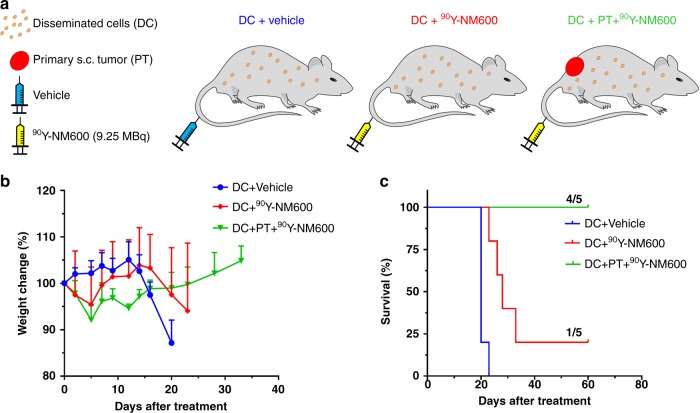
^90^Y-NM600 efficacy in mice models of disseminated EL4 disease. **a** Schematic depiction of the treated cohorts. Mice bearing disseminated (DC) EL4 cells were administered vehicle (DC + Vehicle) or 9.25 MBq ^90^Y-NM600 (DC + ^90^Y-NM600). A third group bearing both DC and a primary s.c. tumor (PT) was injected with 9.25 MBq ^90^Y-NM600 (DC + PT + ^90^Y-NM600). **b** Body weight. Marked weight loss was observed in DC + Vehicle and DC + ^90^Y-NM600 groups indicative of disease progression. **c** Overall survival. Significant extension (*P* < 0.0001) in median survival was observed in both treatment groups with CR achieved in 20% (1/5) and 80% (4/5) of the animals in the DC + ^90^Y-NM600 and DC + PT + ^90^Y-NM600 cohorts, respectively

Figure 5a depicts the generation of clinically relevant models of systemic T cell NHL by administering EL4 cell intravenously, with and without the presence of a localized disease site (s.c. grafts). The ability of ^90^Y-NM600 to achieve high curative rates in these models was tested at a single 9.25 MBq injected activity, which was proven safe and efficacious in the models of localized disease (Figs. 3 and 4). Because systemic tumor burden was difficult to assess, indirect measures of disease progression were employed (Supplementary Table 12). The main symptoms of systemic disease progression were weight loss followed by hind-limb paralysis (Supplementary Movie 1) which indicated the involvement of the peripheral and/or central nervous system (CNS) as a metastatic site^20^. Significant weight loss and paralysis were observed in vehicle-treated animals (*n* = 5) at day 20 post-injection of EL4 cells (Fig. 5b). These effects were significantly (*P* = 0.016) delayed by the injection of 9.25 MBq ^90^Y-NM600 (*n* = 5) resulting in a significant (*P* *<* 0.0001) improvement in median overall survival from 20 to 28 days, in the treated vs. control mice (Fig. 5c). More notably, 20% (1 of 5) of the mice in the 9.25 MBq ^90^Y-NM600 treatment experienced CR at day 60 post-^90^Y-NM600 treatment.

We next evaluated the effects of ^90^Y-NM600 in a mouse model bearing both localized and disseminated lymphoma. Interestingly, when treated animals bore a s.c. EL4 graft, in addition to IV injected cells, mice administered 9.25 MBq ^90^Y-NM600 experienced neither significant weight loss nor paralysis, with 80% showing no signs of tumor burden both systemically and at the primary site (Supplementary Figure 5). Median survival was not reached in this group. The presence of an established tumor site played a significant role in eliciting a swift systemic anti-tumor response that resulted in a high level of CR. Based on this evidence and previous studies showing the stimulation of the immune system by tumor irradiation^21,22^, we hypothesized that ^90^Y-NM600 treatment elicited a potent anti-EL4 immune reaction that resulted in an effective anti-tumor systemic response.

### Immunological effects of ^90^Y-NM600

**Fig. 6:**
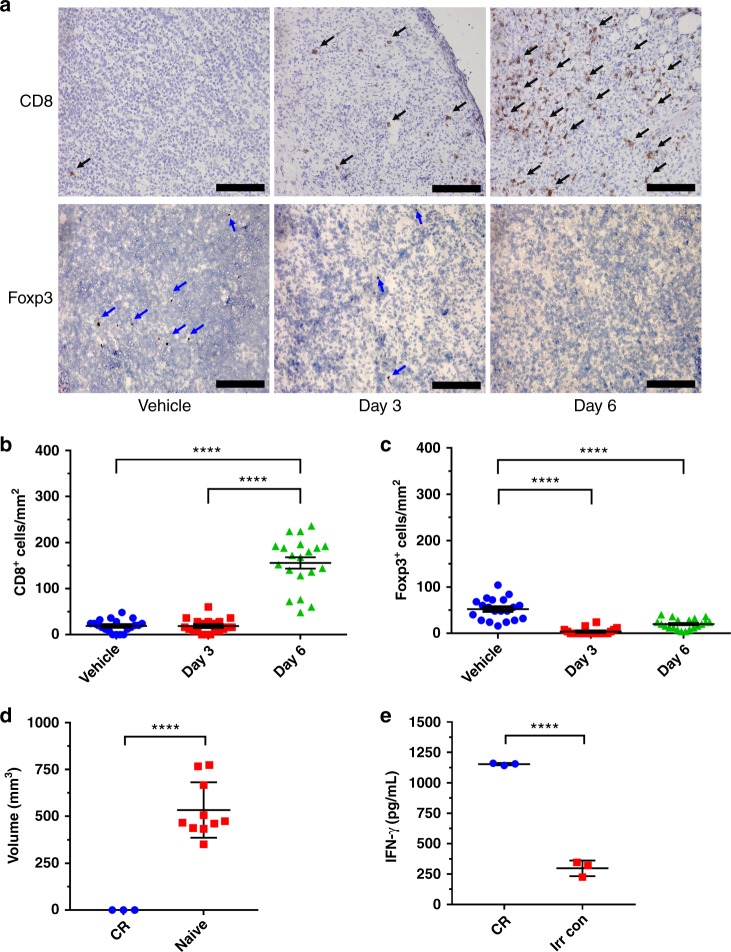
^90^Y-NM600 elicits a T-cell mediated immune response against EL4 tumor. **a** Representative 200x images of CD8 and FoxP3 immunohistochemistry staining of EL4 tumors at days 3 and 6 after ^90^Y-NM600 treatment. Areas of positive staining (brown color) are indicated with arrow heads (blue arrows: CD8 + ; black arrows: FoxP3 + ). Scale bar represents 100 μm. **b**, **c** Quantitative analysis of the stained slides. A significant increase (*P* < 0.0001) in the amount of infiltrating CD8 + T-cells was observed at day 6 post-injection of ^90^Y-NM600. Levels of Foxp3 + cells significantly (*P* < 0.0001) declined in EL4 tumors at days 3 and 6 post ^90^Y-NM600 treatment, compared to vehicle-treated controls. **d** Tumor volume at day 10 after s.c. EL4 cells implantation in naïve vs. CR mice. Naïve mice developed EL4 tumors with 100% penetrance while all CR complete rejected the tumors. **e** ELISA of IFN-γ release by splenocytes coincubated in vitro with EL4 cells. Splenocytes of CR mice showed significantly higher IFN-γ release (*P* < 0.0001) compared to irradiated controls upon stimulation with EL4 cells. Numerical data are presented as mean ± SD

Immunohistochemistry of EL4 tumor sections from vehicle or ^90^Y-NM600-treated mice at days 3 and 6 after injection showed significantly higher levels (*P* < 0.0001) of activated tumor-infiltrating (CD8 + ) T-cells at day 6, compared to mice in the vehicle or day 3 post-treatment groups (Fig. 6a, b). Foxp3 + staining unveiled a significant (*P* < 0.0001) decline in the amount of tumor-associated immunosuppressive regulatory T-cells compared to controls, at both day 3 and 6 after ^90^Y-NM600 injection **(**Fig. 6c**)**. CD4 + staining was inconclusive due to high background expression of CD4 in EL4 tumor cells. The role of immune memory was further corroborated in a tumor re-challenge study where mice who achieved CR from the 9.25 MBq ^90^Y-NM600 treatment cohort were subcutaneously reinoculated with EL4 tumors cells. Figure 6d shows that 10 days after re-inoculation of tumor cells, 100% of naïve mice experienced tumor growth (*n* = 10; ~500 mm^3^) while tumors did not grow in CR mice (*n* = 3). These results indicate the existence of tumor-specific memory that prevented recurrence in the TRT-treated mice. Memory T-cell activation was validated by comparing the levels of IFN-γ release by splenocytes of tumor immunized mice (CR) vs. irradiated control mice, upon co-culture with EL4 cells (Fig. 6e). Significantly higher levels (*P* < 0.0001) of IFN-γ were released by splenocytes of immune mice compared to controls (1153.3 ± 7.7 pg/mL vs. 297.8 ± 52.5 pg/mL) demonstrating the presence of EL4-specific memory T-cells. Overall, these results demonstrated that ^90^Y-NM600 treatment not only elicits CR in mice bearing both disseminated and localized EL4 NHLs but can contribute to a durable memory T-cell response that prevents recurrence.

**Fig. 7:**
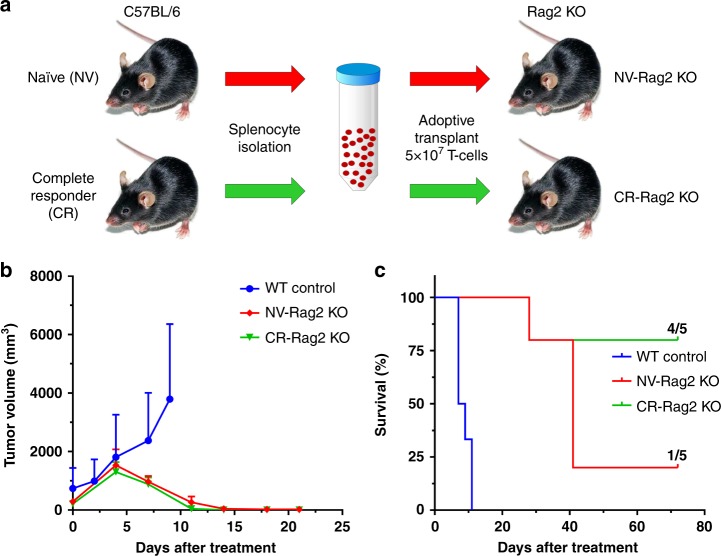
Immunocompetent status affects the levels of complete response to ^90^Y-NM600 treatment. **a** Two groups of immunocompromised Rag2 KO were implanted with EL4 flank grafts then received IV injections of 5 × 10^7^ T-cells from naïve wild-type mice (NV-Rag2 KO) or from wild type mice previously cured with ^90^Y-NM600 (CR-Rag2 KO), followed by a single dose of ^90^Y-NM600. WT control mice were implanted with EL4 cells and received no therapy. **b** Tumor growth curve of Rag2 KO mice bearing EL4 grafts treated with 9.25 MBq ^90^Y-NM600. Similar tumor eradication in both, non-transplanted NV-Rag2 KO and transplanted Rag2 ( + ) groups was observed at day 14 after treatment. **c** Overall survival was higher in CR-Rag2 KO mice, with 80% of the animals alive at day 70 post-injection of ^90^Y-NM600

To further demonstrate the role of the adaptive immune system in the generation of a high level of complete responses after ^90^Y-NM600 treatment, we performed studies in immunocompromised Rag2 KO mice of the C57BL/6 genetic background (Fig. 7a). One group of Rag2 KO mice (NV-Rag2 KO) received (72 h after implanting 10^6^ EL4 cells s.c.) T-cell transplants from naïve wild-type mice. The other group of Rag2 KO (CR-Rag2 KO) mice received (72 h after implanting 10^6^ EL4 cells s.c) T-cell transplants from wild-type mice that were previously cured of EL4 tumors by ^90^Y-NM600 TRT. Both groups of T-cell transplanted Rag2 KO mice (NV-Rag2 KO and CR-Rag2 KO) bearing EL4 s.c. grafts then received ^90^Y-NM600 TRT approximately 10 days after receiving the transplanted cells when the tumors red 700 mm^3^. Unlike EL4-bearing wildtype animals that showed progressive EL4 growth if they received no therapy (no adoptive cells and no TRT), both the NV-Rag2 KO and CR-Rag2 KO experienced significant tumor regression after the administration of a single 9.25 MBq ^90^Y-NM600 dose (Fig. 7b**)**. However, due to a high level of tumor recurrence, only a small fraction of the mice in the NV-Rag2 KO group (20%) showed durable response 60 days after treatment. On the other hand, the CR-Rag2 KO mice which received an adoptive T-cell transplant from CR mice before a single dose of 9.25 MBq ^90^Y-NM600, achieved a markedly higher level of sustained complete response (80%, as in Fig. 7c) that mirrored the sustained response of immunocompetent EL4-bearing mice receiving a comparable dose of TRT (Fig. 7c). These results support the need for a functional immune system and tumor-specific T-cells to attain durable antitumor responses in this model of T-cell NHL.

## Discussion

Radiation therapy has been successfully combined with chemotherapy for the treatment of NHLs; however, efficacy has been limited in patients with high-risk disease. The current limited use of conventional EBRT for the treatment of NHLs is driven by the toxicities associated with the typically large EBRT fields necessary to achieve efficacy, in the context of advanced disseminated disease. On the other hand, TRT offers the advantage of being able to deliver systemic radiation to all disease sites of patients with disseminated or metastatic disease^23,24^. However, effective TRT agents rely on a selective uptake mechanism by tumor cells to achieve a viable therapeutic window. Despite the usage of TRT approaches with radiolabeled antibodies (Bexxar and Zevalin) targeting CD20 receptors overexpressed in B-cell NHLs, similar strategies have not been successful for T-cell NHL mainly due to the lack of known tumor-specific markers and the inability to treat secondary T-cell aplasia resulting from targeting shared antigens with normal T-cells. We have previously demonstrated that radiolabeled APCs selectively accumulate in tumor cells in vivo by exploiting the relative overabundance of lipid rafts in cancer versus normal cells, a mechanism that is ubiquitous to most malignancies^25,26,15,27^. In this study, using the ^86^Y/^90^Y imaging/therapeutic isotopic pair, we employed a theranostic approach to interrogate the in vivo targeting and distribution profile of Yttrium-labeled NM600 noninvasively using PET, to assess the feasibility and inform decisions about the implementation of a ^90^Y-NM600 TRT paradigm in mouse models of localized and/or systemic T-cell NHL.

^86^Y-NM600 showed selective accumulation and prolonged retention in both murine EL4 and human Hut-102 tumor models of T-cell NHL. Importantly, the biodistribution profile of the radiotracer in normal tissue was favorable, exhibiting relatively fast clearance from circulation and gradual body excretion through the hepatobiliary system. Multiple studies including serum stability, in vivo PET/CT imaging, and biodistribution of “free” ^86^Y demonstrated excellent in vivo stability of ^86^Y-NM600 and its tumor specificity. Overall, ^86^Y-NM600 affords desirable characteristics of an imaging probe not only to stratify tumors based on NM600 uptake but to potentially estimate the internal dosimetry and predict the response to ^90^Y-NM600 TRT. Furthermore, given ^18^F-FDG PET/CT is presently used to monitor responses to chemotherapy and radiation in NHL, but only indirectly measures tumor cell metabolism and not truly anti-cancer therapy specific killing, usage of ^86^Y-NM600 to more specifically predict responses to ^90^Y-NM600 TRT may demonstrate clinical utility as a personalized biomarker.

In fact, the elevated ^86^Y-NM600 uptake by EL4 tumors translated into an effective response to TRT treatment. A single intravenous 1.85 MBq ^90^Y-NM600 dose was sufficient to elicit tumor growth delay and extension of survival in mice bearing s.c. EL4 tumors. Activity escalation to 4.63 and 9.25 MBq, which were equivalent to 12.5 and 25 Gy of absorbed dose in the tumor, resulted in significant tumor regression, with the latter achieving CR in a large fraction of the treated animals. These findings parallel those reported by Yoshimoto and colleagues showing similar improved survival in EL4 tumor-bearing mice irradiated with 30 Gy of external beam radiotherapy (EBRT), proving that ^90^Y-NM600 TRT is, at least, as effective as EBRT at controlling the localized disease^28^. Of note is the tumor burden described in those studies which was significantly lower (5-7 fold) than those employed in our work. More importantly, such high tumor absorbed doses were delivered without prohibitive toxicities. Administration of ^90^Y-NM600 at activities up to 9.25 MBq (250 µCi) proved safe, with only transient mild cytopenia, identifying the bone marrow as the potential dose-limiting organ. Long-term adverse effects were not reported in complete responders which showed normal blood CBC, chemistry, and organ morphologies up to 150 days after treatment. Nonetheless, in future planned phase-I dose escalation human trials, the onset of severe myelosuppression as potential dose limiting toxicity will be closely monitored.

We also established the efficacy of ^90^Y-NM600 TRT in a clinically relevant model of metastatic disease. To this end, EL4 cells were injected intravenously with the objective of establishing disseminated disease, which was characterized by weight loss and CNS involvement (hind-limb paralysis) upon progression. Despite a single 9.25 MBq ^90^Y-NM600 injection achieving a significant extension in overall survival, the number of CR in the metastatic model was much lower (1/5) compared to that observed in s.c. graft studies. The incomplete eradication of the disseminated microscopic tumor burden was expected given the suboptimal properties of ^90^Y-NM600 to affect single cells or very small tumors. Due to a long mean free pathway of the β- particles of ^90^Y (3.9 mm), the largest amongst therapeutic beta emitters, local dose deposition is less optimal in small tumors or cell clusters and results in a large fraction of the energy deposited outside the target cells^29^. Such dose deposition effects would be of lesser impact when other radioisotopes with shorter range β- emissions (e.g., ^177^Lu, ^47^Sc, or ^67^Cu) or higher LET (alpha and Auger electron emitters) are employed. The implementation of NM600-based TRT using isotopes better suited for the treatment of microscopic disease such as the alpha-particle emitters ^212^Pb and ^225^Ac will be the subject of follow-up studies.

Interestingly, in the presence of both disseminated disease and an established s.c. primary tumor, a high degree of CR (80%) was recovered with a single 9.25 MBq ^90^Y-NM600 injection, indicating that treatment of the primary tumor graft contributed to a systemic response to TRT. This suggests that TRT is not solely dependent on radiation damage, but also activates immune effector cells capable of inducing a durable systemic anti-tumor response.

Mounting preclinical evidence demonstrates that radiation therapy can induce systemic anti-tumor immunity by modifying the tumor microenvironment (TME) in ways that enhance immune susceptibility. Several mechanisms by which radiation has shown to modulate TME have been identified, including the induction of immunogenic cell death facilitating tumor-neoantigen presentation by antigen presenting cells, or “epitope spreading”, resulting in improved T-cell priming and activation, enhancement of T-cell infiltration through a release of T-cell-attractive chemokines, and/or an upregulation of surface receptors such as MHC-I and Fas that increase vulnerability of cancer cells to cytotoxic CD8 + T-cell attack^30–33^. CD8 + T-cell depletion has resulted in a decreased response to radiation treatment in syngeneic mouse models of NHL, lung carcinoma, and melanoma^28,34,35^. More importantly, a combination of radiation therapy with T-cell-stimulating immunotherapies has been able to achieve swift anti-tumor responses in otherwise immunologically unresponsive cancer models. Preclinical studies have reported a synergistic local and/or abscopal antitumor response to localized EBRT in conjunction with CTLA-4 and/or PD-1/PD-L1 immune checkpoint inhibition (ICI)^21,22,28,36^. These promising results have generated enthusiasm about the combination of EBRT with ICI therapies in the clinical setting^37–39^. However, the potential benefits of local irradiation of tumors in combination with ICI can be affected by the presence of untreated occult sites of disease.

We have previously shown that disseminated TMEs can negatively regulate the immune susceptibility of primary tumor sites remotely (concomitant immune tolerance; CIT) and negate their response to immunotherapies, even after combination with local EBRT irradiation^40^. A conclusion of this study was that CIT could be overcome by irradiating all TMEs, which cannot be achieved using EBRT, at doses required for immunomodulation, without incurring prohibitive systemic toxicities. In this study, we show that our systemic form of radiotherapy, ^90^Y-NM600 TRT, can achieve TME immune modulation and induction of an effective anti-tumor immune response, with limited hematologic toxicity. Tumor-infiltrating T-cells were observed following ^90^Y-NM600 treatment with increased levels of CD8 + lymphocytes and a low abundance of regulatory Foxp3 + T-cells. CD8 + /Foxp3 + cell ratios increased drastically from 0.4 in the vehicle-treated group to 4.8 and 7.7 at days 3 and 6 days post ^90^Y-NM600 injection, respectively, demonstrating the development of an immune susceptible TME following treatment. These conditions were conducive to generating CR observed with disseminated EL4 disease. Rejection of re-inoculated EL4 cells in mice who had undergone CR demonstrated a role of immunologic memory. The significant production of IFN-γ by splenocytes of CR mice upon interaction with EL4 cells suggests this is a T-cell-mediated memory response. We further demonstrated the essential role of the adaptive immune system and T-cells in achieving durable responses to ^90^Y-NM600 treatment using immunocompromised Rag2 KO mice, which lack mature T and B lymphocytes. In these animals, the typical initial EL4 tumor regression was observed following single dose 9.25 MBq ^90^Y-NM600, but most animals (80%) recurred within 60 days post-treatment despite receiving a pre-therapy naïve T-cell transplant. More importantly, a high degree of complete responses (80%) was regained when Rag2 KO mice received transplanted T-cells from CR mice, prior to ^90^Y-NM600 administration. These responses, which were similar to those achieved in immunocompetent mice, confirmed the contribution of tumor-specific T-cells to a durable response and corroborated the presence of memory T-cells in CR immunocompetent mice.

Due to the high sensitivity of lymphomas to radiation treatment (both EBRT and TRT), we realize that the therapeutic efficacy elicited by radiation is maximized in radiosensitive tumors and that TRT alone, or EBRT alone for that matter, is not likely to afford CR in radioresistant tumors. However, we believe that the TME immunomodulatory effects of ^90^Y-NM600 can be extrapolated to other cancer types and leveraged to enhance their responses to ICI or other immune adjuvants. Altogether, our findings suggest that our TRT approach may overcome the limitations of EBRT by delivering radiation to all NHL disease sites with minimal toxicity, which could be of extraordinary value to the treatment of metastatic disease. The induction of a memory T-cell response may contribute to its durability and open the door for combination therapies with immune activating agents. For this purpose, several questions regarding dose, fractionation, timing, radionuclide choice, etc., will have to be addressed to optimize the combination of TRT and immunotherapies.

To our knowledge, this study is the first to implement a successful targeted strategy of radiation therapy for the treatment of T-cell lymphomas. Employing a theranostic approach using the isotopic pair ^86^Y/^90^Y, we demonstrated that ^86^Y/^90^Y-NM600 was selectively taken up by tumors cells while avoiding healthy tissues, affording a therapeutic window that allowed effective anti-tumor response without incurring in significant toxicities. More importantly, anti-tumor effects were observed in both models of localized and disseminated disease. Also, we observed immune effector cell infiltration and activation, leading to durable CR and subsequent generation of anti-tumor immune memory, especially within the context of metastatic/disseminated disease. These findings have implications for the treatment of high-risk T-cell NHL and suggest TRTs, like NM600, may synergize with immunotherapies for various cancer types. Future investigation in clinical trials will be necessary to fully assess the translatability of ^90^Y-NM600 TRT and determine how it best complement the current standard of care for T-cell NHL.

## Methods

### ^86^Y production and radiochemistry

^86^Y (β^+^, *t*_1/2_ = 14.7 h) was produced in a PETrace biomedical cyclotron via irradiation of enriched [^86^Sr]SrCO_3_ (96.4 ± 0.1%) targets with 16.4 MeV protons as described previously^41^. Briefly, irradiated targets were dissolved in 6 N HCl and loaded into a DGA extraction resin. ^86^Y was quantitatively eluted from the column in ~600 µL of 0.1 M HCl. ^90^YCl_3_ was purchased from Perkin Elmer and used without further purification. For the radiolabeling of NM600 with ^86/90^Y, 185-370 MBq (5 -10 mCi) of ^86/90^Y was buffered with 0.1 M NaOAc (pH = 5.5) and 54-81 nmol/GBq (10-15 nmol/mCi) of 2-(trimethylammonio)ethyl(18-(4-(2-(4,7,10-tris(carboxymethyl)-1,4,7,10-tetraazacyclododecan-1-yl)acetamido)phenyl)octadecyl) phosphate (NM600) were added to the mixture. The reaction was incubated for 30 min at 90 °C under constant shaking (500 rpm). The reaction mixture was loaded into an HLB solid phase extraction (Waters) cartridge, washed with 5 mL of H_2_O, and ^86/90^Y-NM600 was eluted in 2 mL of absolute ethanol. The eluate was evaporated under a nitrogen stream, and ^86/90^Y-NM600 was reconstituted in the injection vehicle: normal saline containing 0.4% v/v Tween 20 and sodium ascorbate (0.5% w/v). Radiolabeling yield and radiochemical purity were assessed by instant thin-layer chromatography using silica impregnated paper as the stationary phase and 50 mM EDTA as the mobile phase. iTLC chromatograms were developed using a cyclone phosphor-plate imager reader and analyzed with Optiquant software. ^86/90^Y-NM600 remained at the point of spotting (*R*_f_ = 0) while free ^86/90^Y moved with the solvent front (*R*_f_ = 1). To test the biodistribution of “free” (non-chelated) ^86^Y, an aliquot of ^86^YCl_3_ was buffered with NaOAc (0.01 M) to a final pH of approximately 7.0 an injected as such.

### Serum stability studies

Serum stability of ^86^Y-NM600 was assessed in complete mouse serum by radio-HPLC. Lyophilized mouse serum (Jackson Immunoresearch Labs Inc.) was reconstituted in normal saline (1 mL), spiked with 3.7 MBq of ^86^Y-NM600 and incubated at 37 °C under constant shaking. After 2, 24, and 48 h of incubation, 200 µL aliquots of serum were mixed with an equal amount of acetonitrile and samples were centrifuged at 300 × *g* to remove the precipitated proteins. Supernatants were collected, filtered and analyzed by radio-HPLC. Stability was determined as the ratio of the ^86^Y-NM600 radiopeak area compared to that of metabolites.

### In vitro uptake

In vitro uptake studies were performed in EL4 and normal T-cells by incubating 1 × 10^5^ cells with 37 kBq of ^86^Y-NM600 under regular culture conditions. After an overnight incubation at 37 °C in 5% CO_2_, the cells were spun down, washed twice with 1x PBS, and the cells were harvested and counted in an automated γ-counter (Wizard 2, Perking Elmer, MA). Each condition was done in triplicate.

### Mice and tumor models

All animal studies were performed under the approval of the University of Wisconsin Institutional Animal Care and Use Committee. Female wild type C57BL/6 J (B6) and immunocompromised B6(Cg)-Rag2^tm1.1Cgn^/J (Rag2 KO) mice, which lack mature B and T lymphocytes, were purchased from The Jackson Laboratory and used at 10-12 weeks of age. Male and female NOD.Cg-*Prkdc*^*scid*^ *Il2rg*^*tm1Wjl*^/SzJ (NSG) mice were purchased from The Jackson Laboratory and bred at the University of Wisconsin. Mice were used at 8-12 weeks of age.

EL4 (ATCC, TIB-39), a B6-derived murine T-cell lymphoma cell line, and Hut-102, a human T-cell lymphoma/leukemia cell line, were cultured in RPMI media supplemented with 10% FBS, 1% Penicillin-streptomycin, 1% L-glutamate, 1% HEPES, 1% MEM, 1% Sodium Pyruvate, 0.1% 2 nM Beta-mercaptoethanol, and kept at 37 °C in 5% CO_2_. EL4 cells were inoculated subcutaneously (s.c.) on the flank of B6 mice at 5 × 10^5^ cells/100uL of sterile 1x PBS. Hut-102 tumors were inoculated via s.c. injection on the flank of NSG mice at 10 × 10^6^ cells/100 µL in sterile 1x PBS. Both EL4 and Hut-102 tumor growth were monitored twice weekly by digital caliper measurement. Tumor volume was determined via caliper measurement using the ellipsoid volume formula $\left( {V = L \times \frac{{W^2}}{2}} \right)$, where *L* and *W* are the long and short tumor axis, respectively. In vivo imaging or TRT studies were carried out when the tumor reached a volume of ~700 mm^3^. To establish disseminated disease, either naïve or mice bearing an established EL4 tumor were administered 5 × 10^5^ EL4 cells intravenously in 1x PBS. Disseminated disease progression was monitored by assessing for the development of ascites and by a scoring system based on several clinical symptoms (Supplementary Table 2).

### Imaging and biodistribution

Mice bearing EL4 grafts (*n* = 4) were injected with 9.25 MBq of ^86^Y-NM600 intravenously (lateral tail vein) and sequential CT (80 kVp; 1000 mAs; 220 angles) and static PET scans consisting of 80 million coincidence events (time window: 3.432 ns; energy window: 350-650 keV) were acquired in an Inveon microPET/microCT scanner (Siemens Medical Solutions, Knoxville, TN) at 3, 24, 48, and 72 h post injection of the radiotracer. Prior to each scan, pairs of mice were anesthetized with isoflurane (4% induction; 2% maintenance) and placed in the scanner bed in a prone position. List-mode PET scans were reconstructed using a three-dimensional ordered subset expectation maximization (OSEM3D) algorithm, and the resulting images were fused with the CT images for attenuation correction and anatomical referencing. Region-of-interest analysis of the PET images was performed to determine the magnitude and kinetics of ^86^Y-NM600 uptake in the tumor and normal-tissues of interest. Quantitative data were expressed as percent injected activity per gram of tissue (%IA/g; mean ± SD). A second cohort of mice (*n* = 3) was administered free ^86^Y 9.25 MBq, and PET/CT was performed as described previously. Ex vivo biodistribution was carried out after the final imaging time point at 72 h p.i. of ^86^Y-NM600 of free ^86^Y to corroborate the accuracy of the image-derived quantification and determine additional biodistribution data. Following PET/CT, mice were sacrificed by CO_2_ asphyxiation, EL4 tumors and several normal tissues were collected, wet-weighed, counted in an automated γ-counter (Wizard 2, Perking Elmer, MA), and the %IA/g (mean ± SD) corresponding to each tissue was calculated.

### ^90^Y-NM600 dosimetry estimations

Results from the region of interest (ROI) analysis of the longitudinal PET data were used in conjunction with a standardized MCNP-generated mouse model to estimate ^90^Y-NM600 dosimetry^42,43^. The average activity concentration (%IA/g) within the liver, spleen, kidneys, heart, tumor, bone marrow, and whole-body were scaled with respect to the masses of the organs within the mouse model to compute injected activity (%IA) within each organ. The source-organs that were not delineated were assigned %IA (IA%_src_(*t*)) that were proportional to their organ masses (mass_src_) according to the following equation,

$${\rm{IA}}{\mathrm{\% }}_{{\rm{src}}}(t) = {\mathrm{\% }}{\rm{IA}}_{{\rm{WB}}}(t) \cdot \frac{{{\rm{mass}}_{{\rm{src}}}}}{{{\rm{mass}}_{{\rm{body}}}}},$$

where IA%_WB_(*t*) is the %IA within the whole-body ROI at a given time point, *t*. A piecewise time-integral of IA%_src_(*t*) was used to derive the cumulative activity within each source organ. Between *t* = 0 and the final time point (*t*_final_), a numerical trapezoidal integration method was used; and after *t*_final_, an analytical method of integration was employed assuming a monoexponentially decaying function that was derived by fitting the final two time-points. The standard mouse model, which defines the self-organ and cross-organ energy contributions to target-tissue absorbed dose, converts the cumulative activity within each source organ into the ^90^Y-NM600 absorbed dose per injected activity (Gy/MBq_inj_) within each target organ.

### ^90^Y-NM600 acute toxicity profile in normal mice

Complete blood count (CBC), blood chemistry, and histopathological studies were performed in normal B6 mice to evaluate ^90^Y-NM600 toxicity. Groups of B6 naïve mice (*n* = 5) were administered 1.85, 4.63, or 9.25 MBq of ^90^Y-NM600, or vehicle IV. Regular tail vein bleedings (50 µL) were performed twice weekly for three weeks, then once weekly until day 42 after ^90^Y-NM600 injection, and CBC analysis was performed using a VetScan HM5 veterinary hematology analyzer (Abaxis, Union City, CA). Another cohort (*n* = 9) was injected 9.25 MBq of ^90^Y-NM600; three mice were sacrificed at days 5, 10, and 30 p.i. and 500 µL of blood was collected for a comprehensive blood chemistry panel assay using the VetScan VS2 chemistry analyzer (Abaxis, Union City, CA). Additionally, potential organs at risk for radiotoxicity including the liver, kidneys, spleen, small intestine, and bone marrow were collected, fixed, sectioned, and H&E stained for histopathological analysis. Blood chemistry and histology were compared to control mice.

### Targeted Radionuclide Therapy

After tumors reached a volume of approximately 700 mm^3^_,_ TRT experiments were carried out in randomly selected mice bearing s.c. EL4 tumors. Six groups of mice (*n* = 5-10) received IV a single 1.85, 4.62, 9.25, or 18.5 MBq injection of ^90^Y-NM600, two 4.62 MBq injections of ^90^Y-NM600 9 days apart, or vehicle (*n* = 5). Tumor volume and overall survival were monitored twice a week for 60 days. Humane endpoints were adopted to evaluate moribund mice for euthanasia as per IACUC guidelines. Limited activity, weight loss (20% decrease in weight over a 3-day period), tumor size (>3000 mm^3^), development of palpable ascites, and tumor ulceration were used as moribund endpoints. At day 60 following treatment, half of the mice (*n* = 4) achieving complete tumor remission were immediately re-challenged with EL4 tumor cells, and the other half sacrificed (*n* = 4) at day150 for the assessment of late toxicities. Hematological, liver, and renal toxicity were assessed via CBC analysis, blood chemistry, and hematoxylin and eosin (H&E) staining of the liver, spleen, bone marrow, and kidneys of treated mice, which were compared to age-matched non-treated controls.

### Assessment of immune memory response

To demonstrate immune memory, B6 mice showing durable tumor response by day 60 after treatment were reinoculated with 1 × 10^6^ EL4 cells and monitored for tumor development. Mice showing tumor rejection at day 30 after re-implantation were sacrificed and splenocytes were collected to test for EL4-specific production of interferon gamma (IFN-γ). Single cell splenocyte suspension was prepared by pressing spleens through a 70 µm filter (Corning) while washing with sterile 1x PBS and collecting the filtrate in a 50 ml conical. Cells were spun down, resuspended in 1x PBS, and the splenocytes were counted (Beckman Coulter). IFN-γ production controls were from naïve mice that received a 9.25 MBq ^90^Y-NM600 injection, 30 days prior to splenocyte collection. As previously described^28^, splenocytes were co-cultured with EL4 cells at a 10:1 ratio for 24 h, and IFN-γ levels in the culture supernatant were measured by ELISA assay. Pre-coated ELISA LEGEND Max plates were purchased from Biolegend (San Diego, CA) and used as directed by the vendor.

### Adoptive transplant

Adoptive T-cell transplants were performed by IV injecting 1.5 × 10^8^ splenocytes (~5 × 10^7^ T-cells) isolated from naïve or complete responder (CR) wild-type mice into 10-week-old female Rag2 KO immunocompromised mice, 72 h after s.c. implantation of EL4 cells. TRT studies in these mice proceeded as described above.

### Immunohistochemistry

Mice with established EL4 tumors were treated with either vehicle or 9.25 MBq injection of ^90^Y-NM600. Tumors were harvested on day 0, 3, and 6 after treatment. Tumors were dissected, embedded in OCT medium, slowly frozen over liquid nitrogen, and sectioned. Sectioned slides were fixed in −20 °C acetone for 20 mins, blocked in 5% normal rabbit serum, and labeled overnight at 4 °C using antibody solution. Antibody solutions consisted of a 1:1,000 dilution of anti-FoxP3 (clone FJK-16s, eBioscience), anti-CD8 (clone 53−6.7, eBioscience), anti-CD4 (clone GK1.5, Tonbo Biosciences), or nonspecific isotype control antibody in 5% rabbit serum 1x PBS with 0.01% Triton x-100. Antibody-labeled cells were then detected using ImmPRESS peroxidase secondary and DAB substrate kits (Vector Laboratories). Slides were counterstained with hematoxylin. Quantitative analysis of CD8 + and Foxp3 + cells was carried out by manually counting the amount of positively stained cells in 10 randomly selected 500 µm^2^ fields of view. Bright field image acquisition and cell counting were performed using a BX41 microscope (Olympus, Waltham, MA).

### Statistical analysis

A minimum sample size of *n* = 3 for in vivo PET/CT imaging experiment and *n* = 5 for treatment groups was used. Quantitative data including PET/CT ROI analysis data, biodistribution, CBC, blood chemistry, IFN-γ production, and CD8/Foxp3 counts were expressed as mean ± SD. Comparison between groups was performed via unpaired two-tailed Student’s *T* test. Survival curves were plotted using the Kaplan-Meir method and compared using a Log-rank test. Statistical significance was considered at *P* values of less than 0.05 (****P *<* 0.0001 < ***P < 0.001 < **P < 0.01 < *P < 0.05).

### Reporting summary

Further information on experimental design is available in the Nature Research Reporting Summary linked to this article.

## Supplementary information

Supplementary Information

Description of Additional Supplementary Files

Reporting Summary

Supplementary Movie 1

## Data Availability

Datasets generated or analysed during this study are included in this published article (and its supplementary information files). Additional raw data are available from the corresponding author on reasonable request.
