# Supplementary material for: 90Y-NM600 targeted radionuclide therapy induces immunologic memory in syngeneic models of T-cell Non-Hodgkin’s Lymphoma
Source: Commun Biol. 2019 Feb 26;2:79. doi: 10.1038/s42003-019-0327-4 (PMC6391402; doi:10.1038/s42003-019-0327-4)
Supplement: Supplementary file 1 — Supplementary Information [file 42003_2019_327_MOESM1_ESM.pdf]

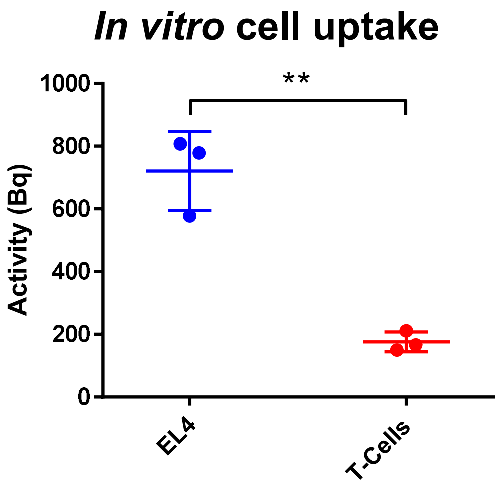

Supplementary Figure 1 *In vitro* uptake of ^{86}Y-NM600 in EL4 cancer cells vs. normal T-cell isolated from the spleens of C57BL/6 mice.

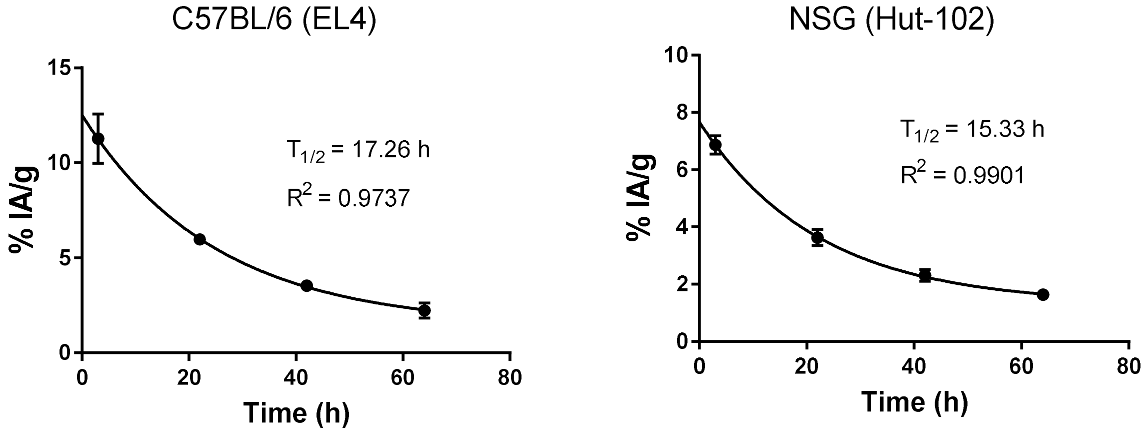

Supplementary Figure 2 Blood time-activity curves of ^{86}Y-NM600 in B6 mice bearing syngeneic s.c. EL4 tumors of NSG mice bearing human Hut-102 T-cell NHL. Blood circulation half-life ($T_{1/2}$) was calculated by fitting the quantitative PET data to a monoexponential decay. Similar circulation $T_{1/2}$ were observed (17.26 h vs. 15.33 h) between the two groups.

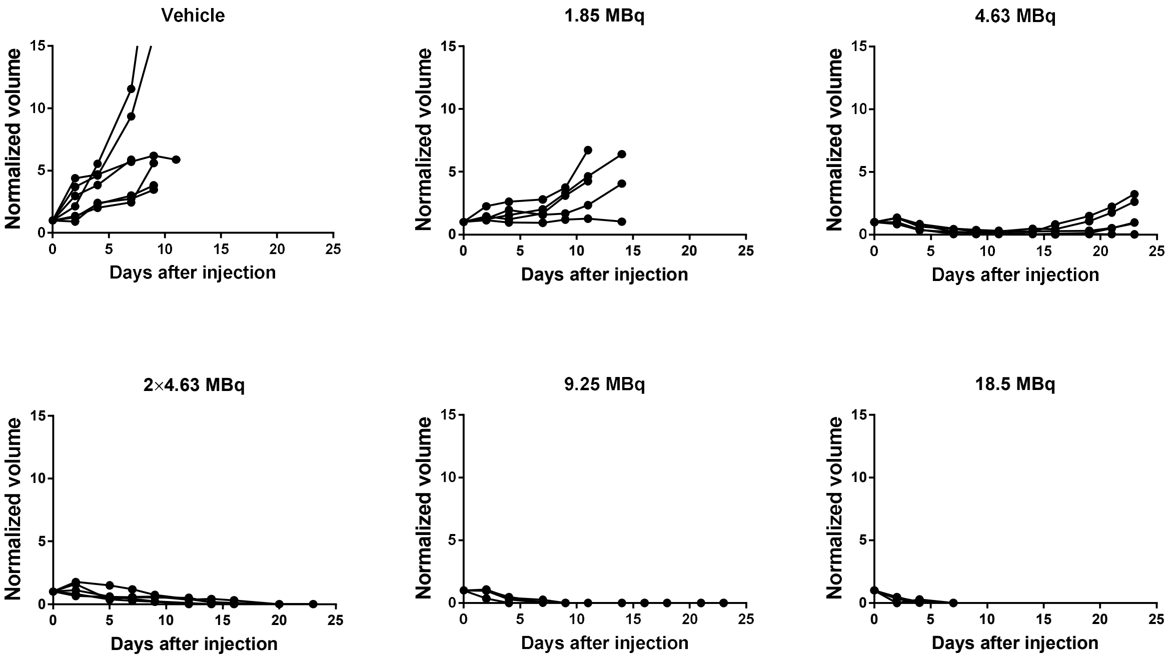

Supplementary Figure 3 Individual tumor growth curves of mice bearing s.c. EL4 tumors injected with vehicle or 1.85, 4.63, 2×4.63, 9.25, or 18.5 MBq of ^{90}Y-NM600. Data are presented as a fraction of the initial tumor volume.

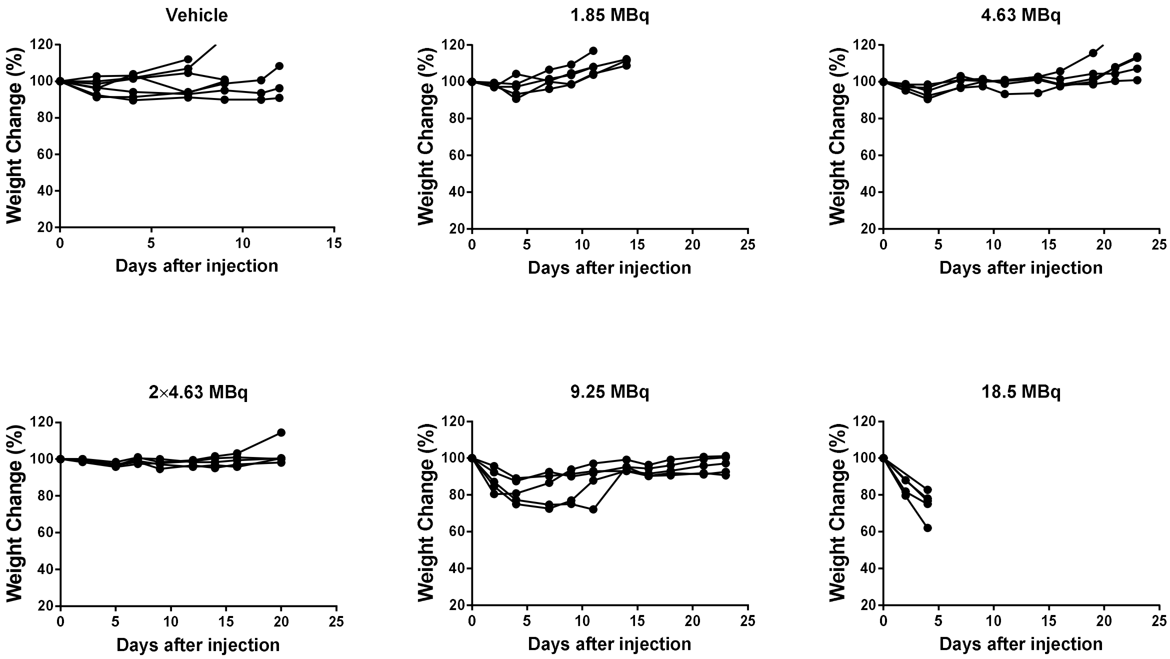

Supplementary Figure 4 Individual weight of mice bearing s.c. EL4 tumors injected with vehicle or 1.85, 4.63, 2×4.63, 9.25, or 18.5 MBq of ^{90}Y-NM600. Data are presents as percent of the initial weight.

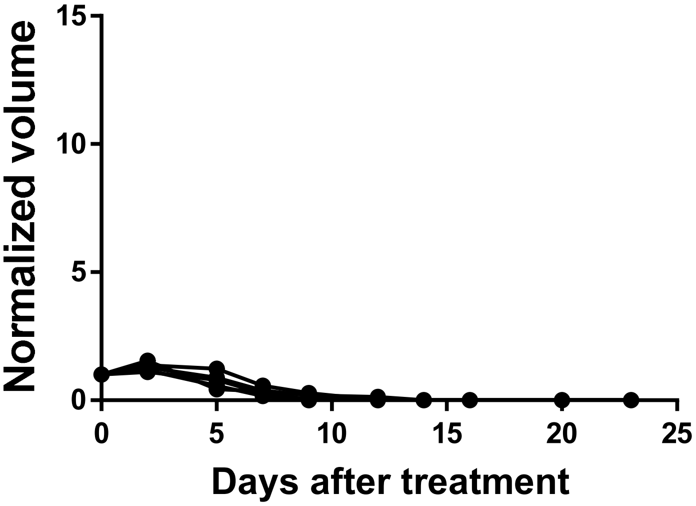

Supplementary Figure 5 Individual tumor growth curves of the primary EL4 tumor in mice bearing both disseminated and localized tumors injected with 9.25 MBq of ^{90}Y-NM600. Data are presented as a fraction of the initial tumor volume.

Supplementary Table 1 Results of the region-of-interest analysis of the longitudinal PET/CT data in tumor-bearing mice administered ^{86}Y-NM600 (9.25 MBq) of free ^{86}Y (9.25 MBq), 68 h post-injection. Data are presented as %IA/g (mean ± SD).

Time (h p.i.)	Tumor (%IA/g)		Heart/blood (%IA/g)		Liver (%IA/g)		Spleen (%IA/g)		Kidneys (%IA/g)		Muscle (%IA/g)		Bone/Marrow (%IA/g)	
	**Mean**	**SD**	**Mean**	**SD**	**Mean**	**SD**	**Mean**	**SD**	**Mean**	**SD**	**Mean**	**SD**	**Mean**	**SD**
**^{86}Y-NM600 in EL4 bearing mice**														
**3**	5.74	0.67	16.45	1.91	13.14	1.25	5.55	1.68	7.79	0.45	2.53	0.08	2.92	0.25
**22**	9.35	0.15	8.71	0.08	13.73	0.64	6.13	0.81	4.48	3.88	1.90	0.00	2.58	0.17
**42**	9.64	1.20	5.16	0.34	10.90	0.86	5.45	0.08	5.74	0.45	1.43	0.03	1.85	0.22
**64**	9.05	1.02	3.26	0.59	8.57	0.95	4.87	1.88	4.62	1.28	1.06	0.12	1.48	0.24
**^{86}Y-NM600 in Hut-102 bearing mice**														
**3**	4.20	0.36	6.87	0.32	6.60	0.62	4.27	0.61	4.00	0.17	1.83	0.32	2.30	0.20
**22**	8.80	1.64	3.63	0.29	8.77	0.68	3.97	2.10	2.97	0.15	0.74	0.07	1.60	0.14
**42**	8.70	0.92	2.30	0.20	7.00	0.61	3.60	0.52	2.43	0.21	0.95	0.08	1.60	0.00
**64**	8.13	1.19	1.63	0.12	5.60	0.80	3.00	1.23	2.07	0.15	0.80	0.04	1.10	0.10
**Free ^{86}Y in EL4 bearing mice**														
**3**	6.67	0.80	2.07	0.21	4.67	0.49	2.40	0.26	5.37	2.30	1.16	0.30	7.70	1.30
**22**	5.90	1.01	1.47	0.21	4.20	0.30	1.80	0.10	3.70	1.10	0.79	0.06	8.03	0.55
**42**	6.05	0.78	1.77	0.61	3.27	1.58	2.50	0.30	3.97	3.33	1.14	0.41	5.30	3.24
**64**	5.70	0.90	1.77	0.47	4.47	0.84	1.87	0.60	4.43	2.87	0.92	0.22	7.57	1.56

Supplementary Table 2 Results of *ex vivo* biodistribution in tumor-bearing mice administered ^{86}Y-NM600 (9.25 MBq) of free ^{86}Y (9.25 MBq), 68 h post-injection. Data is presented as %IA/g (mean ± SD).

	EL4 bearing mice		Hut-102 bearing mice		Free ^{86}Y in EL4 bearing mice	
**Tissue**	**Mean***	**SD**	**Mean***	**SD**	**Mean***	**SD**
Tumor	10.28	0.74	10.54	1.08	4.98	0.42
Blood	2.89	0.33	1.30	0.10	0.02	0.02
Skin	3.88	0.58	1.43	0.08	0.35	0.09
Muscle	0.45	0.10	0.29	0.02	0.10	0.06
Bone/Marrow	1.64	0.33	0.28	0.05	13.90	5.72
Heart	2.09	0.26	1.07	0.06	0.24	0.03
Lung	3.09	0.20	1.40	0.12	0.27	0.08
Liver	9.98	0.47	7.26	0.76	5.14	1.48
Kidney	4.96	0.78	2.45	0.26	4.72	1.80
Spleen	5.26	0.24	3.90	0.71	1.00	0.24
Pancreas	1.20	0.37	0.58	0.11	0.14	0.00
Stomach	1.26	0.18	0.63	0.09	0.41	0.16
Intestine	3.68	0.66	0.60	0.16	0.27	0.09
Brain	0.17	0.04	0.06	0.05	0.02	0.00

*data is presented as percent injected activity per gram of tissue (%IA/g)

Supplementary Table 3 Summary of results from the dosimetry estimation for ^{90}Y-NM600 using longitudinal ^{86}Y-NM600 PET/CT data and The Standard Mouse Model.

Target Organ	Cumulative Activity [(MBq-sec)/MBq]	$\sum_s \boldsymbol{\phi_B}(\boldsymbol{r_t} \leftarrow \boldsymbol{r_s})(\widetilde{\boldsymbol{A}}_s)$	Absorbed Dose (Gy/MBq)	Dose (9.25 MBq)
**Liver**	28644.57	21282.91	3.04	28.1
**Spleen**	1332.53	1044.35	1.74	16.1
**Kidney**	3980.59	3058.51	1.73	16.0
**Lungs**	1361.76	3344.07	3.34	30.9
**Heart**	1943.09	1451.62	1.89	17.5
**Stomach**	1588.72	2322.27	1.99	18.4
**S bowel**	7970.82	10947.58	1.87	17.3
**L bowel**	3014.02	2799.23	1.26	11.7
**Thyroid**	953.23	963.16	1.37	12.7
**Tumor**	26004.45	17978.74	2.69	24.9
**Pancreas**	953.23	662.53	0.95	8.7
**Bone**	998.62	1041.73	1.42	13.1
**Marrow**	30.80	47.16	0.79	7.3
**Carcass**	191699.11	194828.76	1.38	12.8

Supplementary Table 4 Summary of the CBC results for white blood counts (WBC) in 10^3 cells/µL (mean ± SD). N indicates the sample size for each group.

Timepoint (days p.i.)	Control			IA (1.85 MBq)			IA (4.63 MBq)			IA (9.25 MBq)		
	**Mean**	**SD**	**N**	**Mean**	**SD**	**N**	**Mean**	**SD**	**N**	**Mean**	**SD**	**N**
**0**	11.11	1.68	5									
				Acute toxicity study								
**0**				9.05	0.87	5	12.45	5.23	5	12.47	7.94	5
**3**				8.50	4.93	5	8.75	3.83	5	8.86	2.00	5
**7**				8.51	2.48	5	6.33	1.64	5	3.95	1.90	5
**10**				8.33	2.97	5	3.50	1.71	5	1.86	0.71	5
**14**				8.55	2.51	5	5.90	2.13	5	2.73	0.71	5
**17**				8.13	2.82	5	7.35	2.63	5	3.05	1.00	5
**21**				11.06	4.39	5	9.99	2.28	5	7.89	1.33	5
**28**				14.90	5.22	5	14.26	2.15	5	11.16	1.27	5
				Long-term toxicity study								
**150**										14.56	5.94	4

Supplementary Table 5 Summary of the CBC results for lymphocyte counts (LYM) in 10^3 cells/μL (mean ± SD). N indicates the sample size for each group.

Timepoint (days p.i.)	Control			IA (1.85 MBq)			IA (4.63 MBq)			IA (9.25 MBq)		
	**Mean**	**SD**	**N**	**Mean**	**SD**	**N**	**Mean**	**SD**	**N**	**Mean**	**SD**	**N**
**0**	9.54	1.39	5									
				Acute toxicity study								
**0**				8.00	1.06	5	10.87	4.47	5	10.41	6.44	5
**3**				6.23	3.59	5	5.16	2.60	5	5.04	1.54	5
**7**				6.56	1.49	5	3.76	0.95	5	1.65	1.13	5
**10**				6.39	2.19	5	2.32	1.10	5	0.78	0.34	5
**14**				6.51	1.48	5	4.29	1.54	5	1.21	0.30	5
**17**				6.66	1.67	5	5.94	1.60	5	1.71	0.46	5
**21**				8.25	2.33	5	7.78	1.55	5	5.42	1.06	5
**28**				12.29	4.01	5	11.69	2.56	5	6.52	1.99	5
				Long-term toxicity study								
**150**										11.42	5.01	4

Supplementary Table 6 Summary of the CBC results for monocyte counts (MON) in 10^3 cells/µL (mean ± SD). N indicates the sample size for each group.

Timepoint (days p.i.)	Control			IA (1.85 MBq)			IA (4.63 MBq)			IA (9.25 MBq)		
	**Mean**	**SD**	**N**	**Mean**	**SD**	**N**	**Mean**	**SD**	**N**	**Mean**	**SD**	**N**
**0**	0.28	0.18	5									
				Acute toxicity study								
**0**				0.46	0.31	5	0.30	0.36	5	0.56	0.69	5
**3**				0.54	0.42	5	0.07	0.02	5	0.61	0.56	5
**7**				0.44	0.55	5	0.10	0.12	5	0.44	0.22	5
**10**				0.20	0.14	5	0.08	0.07	5	0.12	0.11	5
**14**				0.36	0.36	5	0.11	0.13	5	0.08	0.15	5
**17**				0.39	0.69	5	0.52	0.63	5	0.26	0.18	5
**21**				0.10	0.06	5	0.60	0.46	5	0.30	0.24	5
**28**				0.63	0.33	5	0.69	0.12	5	0.45	0.38	5
				Long-term toxicity study								
**150**										0.64	0.41	4

Supplementary Table 7 Summary of the CBC results for neutrophil counts (NEU) in 10^3 cells/µL (mean ± SD). N indicates the sample size for each group.

Timepoint (days p.i.)	Control			IA (1.85 MBq)			IA (4.63 MBq)			IA (9.25 MBq)		
	**Mean**	**SD**	**N**	**Mean**	**SD**	**N**	**Mean**	**SD**	**N**	**Mean**	**SD**	**N**
**0**	1.29	0.56	5									
				Acute toxicity study								
**0**				0.59	0.40	5	1.28	0.74	5	1.51	1.44	5
**3**				1.73	1.07	5	3.52	2.19	5	3.22	1.15	5
**7**				1.51	1.01	5	2.47	0.89	5	1.86	0.83	5
**10**				1.74	1.17	5	1.09	0.68	5	0.96	0.40	5
**14**				1.69	1.21	5	1.49	0.77	5	1.44	0.59	5
**17**				1.08	0.88	5	1.00	0.51	5	1.08	0.51	5
**21**				2.71	2.21	5	1.61	1.03	5	2.17	0.55	5
**28**				1.99	1.23	5	1.88	0.64	5	4.18	1.61	5
				Long-term toxicity study								
**150**										2.51	1.06	4

Supplementary Table 8 Summary of the CBC results for red blood cell counts (RBC) in 10^6 cells/µL (mean ± SD). N indicates the sample size for each group.

Timepoint (days p.i.)	Control			IA (1.85 MBq)			IA (4.63 MBq)			IA (9.25 MBq)		
	**Mean**	**SD**	**N**	**Mean**	**SD**	**N**	**Mean**	**SD**	**N**	**Mean**	**SD**	**N**
**0**	12.14	0.41	5									
				Acute toxicity study								
**0**				12.80	2.42	5	11.56	0.43	5	11.58	0.60	5
**3**				11.06	1.31	5	11.03	0.54	5	10.99	0.38	5
**7**				11.07	0.64	5	10.70	0.23	5	8.73	0.60	5
**10**				10.61	0.80	5	10.92	0.54	5	9.11	0.64	5
**14**				10.86	0.44	5	10.42	0.21	5	8.89	0.35	5
**17**				10.01	0.95	5	11.31	0.30	5	9.41	1.98	5
**21**				11.00	0.49	5	11.13	0.79	5	10.53	0.55	5
**28**				10.97	1.15	5	11.05	0.45	5	9.79	0.58	5
				Long-term toxicity study								
**150**										9.62	1.01	4

Supplementary Table 9 Summary of the CBC results for hemoglobin concentration (HGB) in g/dL (mean ± SD). N indicates the sample size for each group.

Timepoint (days p.i.)	Control			IA (1.85 MBq)			IA (4.63 MBq)			IA (9.25 MBq)		
	**Mean**	**SD**	**N**	**Mean**	**SD**	**N**	**Mean**	**SD**	**N**	**Mean**	**SD**	**N**
**0**	17.24	1.00	5.00									
				Acute toxicity study								
**0**				16.20	0.97	5	16.48	0.73	5	16.44	0.81	5
**3**				15.66	1.96	5	15.34	0.74	5	15.02	0.76	5
**7**				15.24	0.29	5	14.68	0.47	5	11.28	0.75	5
**10**				15.56	1.58	5	15.28	1.00	5	12.22	0.88	5
**14**				15.34	0.58	5	14.26	0.35	5	12.36	0.69	5
**17**				14.00	1.40	5	15.58	0.47	5	13.90	1.35	5
**21**				15.64	0.92	5	15.90	1.29	5	15.00	0.57	5
**28**				14.74	1.60	5	15.44	0.82	5	14.04	0.84	5
				Long-term toxicity study								
**150**										12.75	1.31	4

Supplementary Table 10 Summary of the CBC results for platelets count (RBC) in 10^3 cells/µL (mean ± SD). N indicates the sample size for each group.

Timepoint (days p.i.)	Control			IA (1.85 MBq)			IA (4.63 MBq)			IA (9.25 MBq)		
	**Mean**	**SD**	**N**	**Mean**	**SD**	**N**	**Mean**	**SD**	**N**	**Mean**	**SD**	**N**
**0**	576	120	5									
				Acute toxicity study								
**0**				151	99	4	609	92	5	742	111	5
**3**				179	115	5	295	112	5	231	82	5
**7**				197	122	5	118	40	5	87	23	5
**10**				128	79	5	149	75	5	98	41	5
**14**				176	137	5	144	110	5	150	71	5
**17**				124	120	5	214	130	5	504	684	5
**21**				112	25	5	197	37	5	192	102	5
**28**				343	113	5	233	63	5	213	51	5
				Long-term toxicity study								
**150**										460.75	128.63	4

Supplementary Table 11 Summary of the blood chemistry panel in EL4 tumor-bearing mice injected with 90Y-NM600 (9.25 MBq) at days 5, 10, 28, and 150 post administration. N indicates the sample size for each group.

	Control (Vehicle)			Day 5 (9.25 MBq)			Day 10 (9.25 MBq)			Day 28 (9.25 MBq)			Day 150 (9.25 MBq)		
Marker	**Mean**	**SD**	**N**	**Mean**	**SD**	**N**	**Mean**	**SD**	**N**	**Mean**	**SD**	**N**	**Mean**	**SD**	**N**
ALB (g/dL)	4.2	0.2	3	4.3	0.2	3	4.8	0.2	3	5.0	0.2	3	3.5	1.0	4
ALP (U/L)	112.3	18.0	3	95.0	6.1	3	88.7	59.2	3	121.3	29.0	3	62.5	28.7	4
ALT (U/L)	33.0	4.0	3	39.3	19.1	3	123.7	114.4	3	42.0	11.8	3	47.8	5.0	4
AMY (U/L)	795.3	30.2	3	779.3	21.0	3	774.7	49.7	3	749.3	64.0	3	865.8	137.8	4
TBIL (mg/dL)	0.3	0.1	3	0.4	0.1	3	0.3	0.1	3	0.3	0.0	3	0.2	0.0	4
BUN (mg/dL)	22.7	2.5	3	25.0	2.6	3	19.7	8.1	3	20.0	1.7	3	21.8	5.5	4
CA (mg/dL)	10.2	0.5	3	9.4	0.2	3	11.5	0.3	3	8.2	5.6	3	10.7	0.4	4
PHOS (mg/dL)	5.5	0.9	3	5.1	0.4	3	14.3	3.9	3	10.8	1.5	3	8.6	1.4	4
CRE (mg/dL)	0.3	0.2	3	0.4	0.2	3	0.5	0.1	3	0.6	0.2	3	0.3	0.1	4
GLU (mg/dL)	285.0	49.4	3	289.3	24.0	3	230.3	78.5	3	238.0	90.1	3	232.0	56.5	4
NA+ (mmol/L)	144.3	1.5	3	144.0	1.0	3	149.7	2.1	3	151.0	1.7	3	152.8	2.5	4
K+ (mmol/L)	4.7	0.5	3	5.0	1.0	3	8.7	0.3	3	7.8	1.0	3	7.8	0.6	4
TP (g/dL)	5.0	0.1	3	4.9	0.1	3	5.5	0.2	3	5.7	0.2	3	5.2	0.3	4
GLOB (g/dL)	0.8	0.1	3	0.6	0.1	3	0.7	0.2	3	0.7	0.1	3	1.7	0.6	4

Supplementary Table 12 Criteria for euthanasia in mice bearing systemic EL4 disease.

	**Grade**				
**Criteria**	**0**	**0.5**	**1**	**1.5**	**2**
Activity	Normal	Mild Decrease	Moderately Decreased	Stationary unless stimulated	Stationary even when stimulated; labored breath.
Weight loss	<10%	10-15%	15-20%	20-25%	25-30%
Hunch	Normal	Hunched when stationary, goes away when active	Small permanent hunch	Moderate permanent hunch	Severe hunch
Appearance	Normal	Slight Ruffle	Moderate ruffle	Ruffle/Greasy	Grizzled/Frizzy
Paralysis	Normal	Slow moving, less tension in tail upon lifting	Partial, mouse starts scratching legs	One leg completely paralyzed, other has partial mobility	Both legs are completely paralyzed (dragging)

-Euthanize if mouse reaches a total score of 4 or higher
-Euthanize if mouse reaches 2 on paralysis or activity or if they surpass 25% weight- loss
