# Supplementary material for: 90Y-NM600 targeted radionuclide therapy induces immunologic memory in syngeneic models of T-cell Non-Hodgkin’s Lymphoma
Source: Commun Biol. 2019 Feb 26;2:79. doi: 10.1038/s42003-019-0327-4 (PMC6391402; doi:10.1038/s42003-019-0327-4)
Supplement: Supplementary file 2 — Description of Additional Supplementary Files [file 42003_2019_327_MOESM2_ESM.docx]

**Supplementary Movie 1** Clinical manifestation of disseminated EL4 disease. Symptomatic mice show bilateral hindlimb paralysis indicating peripheral nervous system involvement.
